# Organomineral fertilizer application enhances *Perilla frutescens* nutritional quality and rhizosphere microbial community stability in karst mountain soils

**DOI:** 10.3389/fmicb.2022.1058067

**Published:** 2022-11-24

**Authors:** Ying Li, Qi Shen, Xiaochi An, Yuanhuan Xie, Xiuming Liu, Bin Lian

**Affiliations:** ^1^State Key Laboratory of Environmental Geochemistry, Institute of Geochemistry, Chinese Academy of Sciences, Guiyang, China; ^2^College of Life Sciences, College of Marine Science and Engineering, Nanjing Normal University, Nanjing, China; ^3^Institute of Medical Plant Physiology and Ecology, School of Pharmaceutical Sciences, Guangzhou University of Chinese Medicine, Guangzhou, China

**Keywords:** karst soil, organomineral fertilizer, *Perilla frutescens*, nutritional quality, rhizosphere microbial community

## Abstract

**Introduction:**

Applications of organomineral fertilizer (OMF) are important measures for developing organic agriculture in karst mountain areas. However, the influence of OMF on the structure and function of soil microbial diversity and their relationship with crop yield and quality are still unclear.

**Methods:**

Based on soil science, crop science, and high-throughput sequencing methods, we investigated the changes of rhizosphere soil microbial communities of *Perilla frutescens* under different fertilization measures. Then, the relationship between *P. frutescens* yield and quality with soil quality was analyzed.

**Results:**

The results showed that the addition of OMF increased the amount of total carbon and total potassium in soil. OF, especially OMF, improved *P. frutescens* yield and quality (e.g., panicle number per plant, main panicle length, and unsaturated fatty acid contents). Both OF and OMF treatments significantly increased the enrichment of beneficial microorganism (e.g., *Bacillus*, *Actinomadura*, *Candidatus_Solibacter*, *Iamia*, *Pseudallescheria*, and *Cladorrhinum*). The symbiotic network analysis demonstrated that OMF strengthened the connection among the soil microbial communities, and the community composition became more stable. Redundancy analysis and structural equation modeling showed that the soil pH, available phosphorus, and available potassium were significantly correlated with soil microbial community diversity and *P. frutescens* yield and quality.

**Discussion:**

Our study confirmed that OMF could replace *CF* or common OF to improve soil fertility, crop yield and quality in karst mountain soils.

## Introduction

Application of chemical fertilizers (CFs) can significantly increase soil fertility and crop yield in a short time. However, long-term excessive application of CFs damages soil microbial communities and biological activities, which results in decreased soil quality, increased dependence of crop growth on fertilizer nutrients, and aggravated agricultural surface source pollution (Gomiero et al., 2011; Kour et al., 2020; Ren et al., 2020). To reduce the negative effects caused by excessive application of CFs, organic fertilizers (OFs) are usually used to replace or partially replace CFs to protect soil biodiversity and maintain soil ecological balance (Megali et al., 2013; Steffen et al., 2015). Increased OFs application can increase soil carbon storage and plant nutrients, and improve soil biological activity, which are of great significance for mitigating climate warming and developing sustainable agricultural production (Gattinger et al., 2012; Seufert et al., 2012). Many studies have shown that OFs application is an effective way to improve crop yield and quality (Liu et al., 2020, 2021; Du et al., 2022), and increase soil microbial richness and diversity (Zhou et al., 2015; Cui et al., 2018; Li et al., 2020). However, the contents of mineral nutrients such as nitrogen, phosphorus, and potassium in OFs are low, and the increased amount of fertilizer needed increases the cost and application difficulty. Therefore, it is very necessary to improve the contents of mineral elements in traditional OFs.

As a new fertilizer combining the advantages of organic fertilizer and inorganic fertilizer, the nutrient release effect of OMFs occurs simultaneously with the crop growth process, making the agronomic efficiency higher when compared with the inorganic fertilizer (Kiehl, 2008). Compared with CFs, OMFs can reduce the loss of some nutrients, such as nitrogen volatilization, phosphorus fixation and potassium leaching (Aguilar et al., 2019). Compared with common OFs, OMFs are rich in mineral elements necessary for crop growth. OMFs are usually composed of natural organic matter sources and inorganic element sources. Organomineral fertilizer ingredients are mostly agricultural wastes such as chicken manure, coffee shell, wood waste, sewage sludge and sugarcane cake, combined with urea, calcium superphosphate, potassium chloride, magnesium silicate, calcium sulfate and other inorganic chemical fertilizers (Efanov et al., 2001; Carvalho et al., 2014; Grohskopf et al., 2019; Gonçalves et al., 2021; Hawrot-Paw et al., 2022; Ngo et al., 2022). According to the published articles, the research on the application effect of organic mineral fertilizer mainly focuses on the agronomic efficiency of crops. Compared with the crop response to inorganic fertilizers, the response to organomineral fertilizers is quite variable. The reported results are gains (Efanov et al., 2001; Deeks et al., 2013; Carvalho et al., 2014; Sakurada et al., 2016; Vollú et al., 2018; Ngo et al., 2022), losses (Antille et al., 2017; Frazão et al., 2019), or equivalent efficiency (Corrêa et al., 2018; Dias et al., 2020; Mumbach et al., 2020). Those varied results may be related to the different ingredients of OMFs, the amount of fertilizer applied and the environment of the study sites. Limited research literature showed that OMF, as a source of organic carbon and mineral elements, had a positive effect on the quantity and activity of soil microorganisms (Hawrot-Paw et al., 2022), but had little effect on the rhizosphere bacterial diversity of crops (Vollú et al., 2018).

The staggering production of cuttings and quarry by-products from mining activities results in a huge environmental burden; however, the combined use of these cuttings and low-grade mineral rocks can help reduce this pollution (Basak et al., 2021; Syed et al., 2021). The OMF formed by the combination fermentation of low-grade mineral powder and agricultural waste can more fully reflect the production concept of energy saving and environmental protection, and reduce the cost in the production process of inorganic fertilizers such as urea and phosphate fertilizer. Previous studies have shown that OMF produced by mixing rock powder containing potassium and phosphate with OF or mixing rock powder during OF fermentation can provide beneficial mineral nutrients for crop growth without introducing toxic heavy metal pollution (Theodoro and Leonardos, 2006; Biswas et al., 2009; Lian et al., 2020; Basak et al., 2021; Syed et al., 2021). As an important support of soil quality, soil microorganisms are sensitive to fertilization managements (Wang et al., 2020; Bello et al., 2021). However, there have been no reports on the effects of OMFs application on soil microbial community diversity and composition in karst mountain soils.

*P. frutescens* is an annual herb in the Labiaceae family and is a widely cultivated cash crop in Asian countries (Hu et al., 2010; Yu et al., 2017). In China, it has traditionally been used in medicine and food, and has been cultivated for more than 2000 years (Lee and Kim, 2007). The oil-rich seeds of *P. frutescens* are used to make condiments in traditional Asian cuisines (Luitel et al., 2017). As the raw material of cooking oil, perilla grains are rich in unsaturated fatty acids and have a high content of ɑ-linolenic acid, up to 50–70%; this is the highest ɑ-linolenic acid content known in plants (Yu et al., 2017). In addition to value as a food item, *P. frutescens* is also used in traditional Chinese medicine, and as food decoration and a coloring agent (Tian et al., 2014). Moreover, *P. frutescens* is an important agricultural crop in karst areas of southwest China (Tian et al., 2017).

Potassium is one of the main elements necessary for plant growth. According to the standard of the Second Soil Survey of China (China Soil Science Database^1^), the soil in karst areas is in a state of potassium deficiency (total *K* < 10 g·kg^−1^ and available *K* < 100 mg·kg^−1^). Therefore, potassium supplementation is necessary to improve soil fertility in karst areas of southwest China. For this reason, our OMF was fermented from potassium-containing rocks (potassium feldspar) together with agricultural wastes such as chicken manure and straw. We hypothesized that the application of potassium-containing OMF was beneficial to improving *P. frutescens* quality and yield, and had positive effects on the distribution and structure of soil microbial communities. To test this hypothesis, the yield, quality, and rhizosphere soil microbial community characteristics of *P. frutescens* were analyzed and studied based on soil science, crop science, and high-throughput sequencing technology methods. The aim of this study was to reveal: (1) differences in soil microbial diversity and community structure of the *P. frutescens* rhizosphere under different fertilization treatments; (2) the relationship among *P. frutescens* yield and quality, soil characteristics, and microbial community under different fertilization treatments; and (3) the effects of OMF on soil and crop quality. This study investigated for the first time the feasibility of using OMFs to improve the quality of *P. frutescens* and the abundance of soil beneficial microbial community in the potassium-deficient karst areas. The results will be conducive to the application of new OMFs in karst areas, and improve the yield and quality of *P. frutescens* which is widely cultivated in this area.

## Materials and methods

### Experimental design and sample collection

The experimental field site was located in Changzhai Village, Changshun County, Guizhou Province, China (26°01′25″ N, 106°30′55″ E; elevation, 1,004 m). The strata are mainly light-colored limestone of the Permian Qixia Formation and Maokou Formation. The soil type is yellow soil according to the Chinese soil classification system (Gong, 1999) and Orthic Acrisols according to the World Reference Base (WRB) soil classification system (USS Working Group WRB, 2015). The region has a subtropical monsoon humid climate. The annual average temperature is 15.1°C, the annual average precipitation is 1396.7 mm, the annual sunshine duration is 1202.1 h, and the annual frost-free period is 275 days.

Four treatments were set up in the field experiment: blank control group (CK), no fertilization; *CF* group, compound *CF* was applied; OF group, ordinary OF was applied; OMF group, potassium-containing OMF was applied. Four parallel plots (5 m × 5 m) were set for each treatment group (total, 16 plots). The randomized complete block design was used to divide the sample area blocks (Supplementary Figure S1). On 7 May 2019, *P. frutescens* was planted and fertilized. The *P. frutescens* seeds were the 1^st^ generation hybrids cultivated by the Oil Materials Research Institute of Guizhou Academy of Agricultural Sciences (China).

The fertilizer specifications used were as follows:

The raw materials for OF fermentation were mushroom residue, distiller’s grains, straw, and chicken manure, which were mixed according to a mass ratio of 1:1:1:2 and EM microbial agent was added for fermentation (1 kg bacterial agent was added for every 10 T substrate). The mixture was then fermented in a fermentation tank for 30 days with periodic stirring. The total nutrient content (N + P_2_O_5_ + K_2_O) was 7.2%, among which the content ratio of N:P_2_O_5_:K_2_O was 0.9%:2.9%:3.4%, organic matter was 48.3%, and pH was 7.7.

Potassium-containing OMF was made by mixing the raw fermentation materials of the OF and potassium-containing rock powder, which contained 76% potassium feldspar, and passing the mixture through a 2-mm sieve. In accordance with a mass ratio of 3:1, the chemical composition was as follows: Al_2_O_3_, 17.11%; SiO_2_, 54.06%; K_2_O, 9.09%; CaO, 1.9%; Fe_2_O_3_, 6.15%; and MgO, 3.41% (Sun et al., 2019). Then, EM microbial agent was added for fermentation (1 kg bacterial agent per 10 T substrate). The raw materials were thoroughly mixed and fermented for 30 days with periodic stirring. The total nutrient content of OMF was 6.4%, with a content ratio of N: P_2_O_5_: K_2_O of 1.6%:1.1%:3.7%, the content of organic matter was 56.4%, and the pH was 7.6. OF and OMF were produced by Guizhou Guifu Ecological Fertilizer Co., LTD. (China).

The compound *CF* was produced by Guizhou Xiyang Fertilizer Co., LTD. (China) and had a total nutrient content ≥45%, N:P_2_O_5_:K_2_O content ratio was 15%:15%:15%. Urea was produced by Guizhou Chitianhua Tongzi Chemical Co., LTD. (China) with a total N ≥ 46.4%. Phosphate fertilizer was produced by Fuda Phosphorus Chemical Co., LTD. (China) with P_2_O_5_ ≥ 12%. The proportions of N, P, and K in OF and OMF groups were balanced by urea and phosphate fertilizer. All treatment groups had fertilizer applied with a content ratio of N:P_2_O_5_:K_2_O of 15:15:15, and application amounts are shown in Table 1.

**Table 1:** Fertilization doses of different treatment groups.

Treatments	Base fertilizer				
	Inorganic compound fertilizerkg.hm^−1^	Conventional organic fertilizerkg.hm^−1^	Organomineral fertilizerkg.hm^−1^	Urea (N)kg.hm^−1^	Calcium superphosphate (P_2_O_5_)kg.hm^−1^
CK	0	0	0	0	0
*CF*	225.00	0	0	0	0
OF	0	993.35	0	53.48	40.37
OMF	0	0	912.47	41.28	197.55

CK is the control blank group; CF is conventional inorganic compound fertilizer group; OF is conventional organic fertilizer group; OMF is organomineral fertilizer group.

Rhizosphere soil sampling was conducted on 19 August 2019, which was the 105^th^ day of *P. frutescens* growth. Rhizosphere soil samples were arbitrarily collected from eight *P. frutescens* plants in each plot and mixed into one sample. A total of 16 mixed soil samples were collected. When collecting rhizosphere soil, the whole plant was first dug up, and the scattered soil at the root was gently shaken off. The remaining soil attached to the root system was considered the rhizosphere soil (Sun et al., 2021). The soil samples were frozen and transported to the laboratory on dry ice, passed through a 2-mm sterile sieve, and the plant roots were removed. Each sample was divided into two parts: one part was naturally air-dried to determine physicochemical properties, and the other was stored in a − 80°C freezer for DNA extraction.

### Soil physicochemical analysis

Soil pH was analyzed by vibrating slurry with a water:soil ratio of 2.5:1 (v/w) and determined using a pH meter (Mettler-Toledo FE28, Switzerland; Marcos et al., 2019). Soil total organic carbon (TOC), total organic nitrogen (TON), total carbon (TC), and total nitrogen (TN) were determined using an elemental analyzer (Vario MACRO Cube, Germany; Liu et al., 2014). Total phosphorus (TP) and total potassium (TK) were determined by sodium hydroxide melting flame spectrophotometry (Ren et al., 2016). Available phosphorus (AP) was determined by the NaHCO_3_ method, and available potassium (AK) was determined by ammonium acetate extraction–flame spectrophotometry (Lu, 1999).

### *P. frutescens* yield and quality analysis

On 21 September 2019, *P. frutescens* were harvested on the 137th day of growth. The *P. frutescens* yield was measured after harvest. The main indicators were plant height, biomass per plant, number of stem nodes, number of effective branches at one time, length of effective branches at one time, number of branch angles, number of panicles per plant, length of the main panicle, number of fruits in a single row of the main panicle, number of fruits on the main ear, and number of grains in the 10 main ears. After the perilla seeds were harvested, seed quality was inferred by determining fatty acid content (e.g., palmitic acid content, stearic acid content, oleic acid content, linoleic acid content, and linolenic acid content), total lipid content, and crude protein content. The fatty acid content was determined by gas chromatography–mass spectrometry (GCMS-QP2010, Schimadzu, Japan; Javier et al., 2018; Ko et al., 2018). Fat content was determined by sequential Soxhlet extraction according to ISO 659:2009 (Kourimska et al., 2018). The crude protein content was determined by the Kjeldahl method, the nitrogen concentration of the sample was calculated with a conversion factor (6.25), and the total nitrogen and protein mass were determined to obtain the crude protein content (Yaldiz and Camlica, 2020).

### DNA extraction and high-throughput sequencing

Total soil DNA was extracted from 0.5 g soil according to the manufacturer’s instructions for the E.Z.N.A.® Soil DNA Kit (Omega Bio-Tek, USA). The DNA extraction quality was detected using 1% agarose gel electrophoresis, and the DNA concentration and purity were determined using a NanoDrop2000 spectrophotometer (Thermo Fisher Scientific Co., LTD., USA). Using the extracted DNA as a template, the V3–V4 region of the bacterial 16 s rRNA gene was amplified using the primers 338F (5′-ACTCCTACGGGAGGCAGCAG-3′) and 806R (5′-GGACTACHVGGGTWTCTAAT-3′; Xu et al., 2016). The fungal ITS region was amplified using the primers ITS1F (5′-CTTGGTCATTTAGAGGAAGTAA-3′) and ITS2R (5′-GCTGCGTTCTTCATCGATGC-3′; Adams et al., 2013). PCR amplification conditions and high-throughput sequencing were conducted as described in Li et al. (2021). Sequencing was performed on Illumina’s MiSeq PE300 platform (Shanghai Majorbio Bio-pharm Technology Co., LTD., China). The raw sequence data reported in this paper were deposited in the NCBI SRA database (serial numbers: bacteria, PRJNA836163; fungi, PRJNA836186).

Trimmomatic (version 0.33^2^) was used for quality control of the original sequences (Bolger et al., 2014), and FLASH (version 1.2.11^3^) was used for splicing (Magoc and Salzberg, 2011). The splicing sequence data were analyzed using UPARSE (version 7.1;^4^ Edgar, 2013), and sequences with a similarity of ≥97% were assigned to the same operational taxonomic unit (OTU). After quality control and concatenation of the original sequences of all samples, 1,088,672 and 976,526 high-quality sequences of bacteria and fungi were obtained, respectively. At the 97% sequence similarity level, the sequences clustered into 5,685 and 2,585 OTUs, respectively. For each representative sequence, the SILVA (bacteria^5^) and UNITE (fungi^6^) databases were used to annotate taxonomic information (Fan et al., 2020; Kang et al., 2022).

### Statistical analysis

Mean value, standard deviation, and variance analysis of soil physicochemical properties and *Perilla* yield and quality were analyzed using Microsoft Excel 2010 and SPSS Statistics (version 20.0, IBM, USA). Differences between mean values were determined by one-way ANOVA and LSD post-hoc test (*p* < 0.05). All bioinformatic analyses were performed using R (version 3.6.1; https://cran.r-project.org/bin/windows/base/old/3.6.1/).

The alpha diversity (Sobs, Chao, and Shannon indices) of *Perilla* rhizosphere soil microbial communities was estimated based on OTUs. All indices were calculated by the “vegan” (Dixon, 2003) and “picante” (Kembel et al., 2010) packages in R (version 3.6.3). The linear discriminant analysis (LDA) effect size (LEfSe) method was used to assess potential bacterial and fungal biomarkers (from phylum to genus) within soil microbiomes that were specifically enriched under different fertilization management types based on *p* < 0.05 and an LDS score > 4.0 (Segata et al., 2011). Principal coordinates analysis (PCoA) was performed to calculate the gradient of compositional changes for bacterial and fungal microbial communities (based on Weighted-Unifrac distance matrix) using the ggplot2 package (Lozupone and Knight, 2005). Differences in bacterial and fungal communities between different samples were analyzed by Adonis test.

After variance inflation factor (VIF) analysis, pH, TC, TOC, TN, TK, AP, and AK with a VIF threshold less than 10 were selected for redundancy analysis (RDA) between environmental factors and soil microbial communities. Variance partitioning analysis (VPA) was used to quantitatively evaluate the individual and common explainability of environmental factor variables for microbial community differences. In addition, FAPROTAX (Louca et al., 2016) and FUNGuild (Nguyen et al., 2016) were used to analyze the ecological functions of soil bacteria and fungi, respectively. Kruskal–Wallis H test was used to test the significance of differences between groups.

### Co-occurrence network analysis

To study the effect of different fertilization management techniques on the relationship of soil microbial communities, soil bacterial and fungal communities were combined based on fertilization management technique, and a soil microbial co-occurrence network based on genus classification was constructed. The co-occurrence network was constructed with genera that had a relative abundance greater than 0.1% based on random matrix theory (Deng et al., 2012). To simplify the networks for better visualization, a Spearman’s correlation between the two genera was considered statistically significant if the Spearman’s correlation coefficient (*r*) was >0.6 and the *p* value was <0.05. Moreover, *p* values were adjusted using the Benjamini–Hochberg FDR method (Benjamini and Hochberg, 1995). Spearman’s correlation and network attributes were calculated using the WGCNA, Psych, Igraph, and fdrci packages in R (version 3.6.3) (Csardi and Nepusz, 2006). The network attributes included the number of edges, average clustering coefficient, average degree, modularity, average path length, graph density, and betweenness centrality. Higher numbers of nodes and edges, graph density, average degree, and lower average path length indicate a more complex and connected network (Ma et al., 2016; Jiao et al., 2021). The higher the betweenness centrality value of microbial species, the greater the critical role of the species in the network. The Fruchterman–Reingold layout algorithm was used in the interactive platform Gephi (version 0.9.2^7^) for network visualization and network topology parameter calculation (Bastian et al., 2009). The network stability was evaluated by removing the nodes in the static network to estimate the speed of robustness decline, and the network robustness was evaluated by the natural connectivity of the nodes (Yuan et al., 2021; Zhu et al., 2022).

### Structural equation model analysis

A structural equation model (SEM) was used to identify the direct and indirect effects of soil physicochemical properties (such as pH, TC, AK, and AP) on bacterial and fungal diversity (Shannon index), and *P. frutescens* yield (biomass per plant) and quality (linoleic acid content). To reduce SEM complexity, the representative indices of soil physicochemical properties were calculated by PCoA (Sun et al., 2021). All variables were standardized using Z-transformation (mean = 0, standard deviation = 1; Du et al., 2022).

The theoretical model assumed that: (1) soil pH has a direct impact on soil nutrient content, microbial community diversity, and *P. frutescens* quality or yield, (2) the soil available phosphorus, potassium, and total carbon had direct or indirect effects on the *P. frutescens* quality and yield and the soil microbial community, and (3) the *P. frutescens* yield has a direct effect on the soil microbial community. Model fitting was performed using root mean square error of approximation (RMSEA), probability level *p* value, Bentler comparative fit index (CFI), maximum likelihood goodness of fit (*χ*^2^), and degrees of freedom (df) tests (Du et al., 2022). The SEM was constructed using Amos Graphics (version 24.0, IBM Corp., USA; Liu, L. et al., 2019).

## Results

### Soil physicochemical properties

Compared with the CK group, the OMF group significantly increased soil TC and TK contents (*p* < 0.05; Table 2). Fertilization also significantly increased soil AK content (*p* < 0.05; Table 2). In addition, there were no significant differences in the physicochemical properties between the different fertilization treatment groups. The measurement results of different fertilization treatment groups indicated that short-term fertilization treatments may not significantly improve soil physicochemical properties. However, numerical analysis demonstrated that OF and OMF treatments tended to produce better results than no fertilization and *CF* treatment.

**Table 2:** Soil chemical properties under different fertilization treatments.

Chemical factor	CK	*CF*	OF	OMF
pH	5.98 ± 0.19a	5.91 ± 0.07a	6.04 ± 0.10a	6.00 ± 0.13a
TOC	15.73 ± 0.79a	16.12 ± 0.72a	16.31 ± 0.78a	16.49 ± 1.14a
TON	0.14 ± 0.01a	0.15 ± 0.01a	0.15 ± 0.01a	0.15 ± 0.01a
TC	20.43 ± 0.50b	20.78 ± 0.66ab	20.86 ± 0.29ab	21.16 ± 0.34a
TN	1.92 ± 0.06a	1.97 ± 0.05a	1.95 ± 0.04a	1.93 ± 0.03a
TP	0.80 ± 0.05a	0.81 ± 0.04a	0.82 ± 0.04a	0.81 ± 0.04a
TK	5.59 ± 0.14b	5.68 ± 0.20ab	5.70 ± 0.19ab	5.84 ± 0.09a
AP	28.51 ± 6.65a	35.24 ± 5.88a	30.89 ± 5.73a	30.42 ± 5.61a
AK	92.75 ± 20.35b	99.5 ± 44.44a	98.50 ± 31.29a	109.75 ± 30.83a

pH stands for soil pH; TOC represents soil total organic carbon content, g·kg^−1^; TON stands for soil total organic nitrogen content, g·kg^−1^; TC stands for soil total carbon content, g·kg^−1^; TN stands for soil total nitrogen content, g·kg^−1^; TP represents soil total phosphorus content, g·kg^−1^; TK represents soil total potassium content, g·kg^−1^; AP stands for soil available phosphorus content, mg·kg^−1^; AK stands for soil available potassium content, mg·kg^−1^. Different letters (a, b) on the same row indicate values that are significantly different (p < 0.05) based on one-way ANOVA and LSD post-hoc test.

### *P. frutescens* yield and quality

The analysis of *P. frutescens* yield during the harvest period showed that, compared with the CK group, the *CF*, OF, and OMF groups all significantly improved the yield indicators, including the number of stem nodes, number of effective branches at one time, and number of branch angles. However, there were no significant differences among the three fertilization treatments (Table 3). In addition, the number of panicles per plant and length of the main panicle in the OMF group were significantly greater than those in the other treatment groups.

**Table 3:** *Perilla frutescens* yield indexes under different fertilization treatments.

Fertilizer regimes	Individual plant biomass (g)	Plant height (cm)	Nodes on main stem	Number of once effective branches	Once effective branch length	Number of pod angles on branches	Spike number per plant	Length of main spike (cm)	Number of main spike per row	Number of pod horns in main ear	Grain number of 10 main spike
CK	365.56 ± 77.36a	130.81 ± 8.80a	10.06 ± 1.00b	18.81 ± 2.34b	109.06 ± 11.39a	26.25 ± 4.28b	51.31 ± 10.93c	27.56 ± 2.63b	32.13 ± 2.80a	128.50 ± 11.21a	35.94 ± 3.38ab
*CF*	383.31 ± 64.18a	130.88 ± 9.14a	10.69 ± 0.60ab	20.94 ± 1.24a	110.13 ± 11.63a	28.44 ± 3.52ab	55.25 ± 11.53bc	27.13 ± 2.19b	31.88 ± 1.59a	127.50 ± 6.35a	35.56 ± 2.85b
OF	433.31 ± 163.47a	134.25 ± 12.08a	10.63 ± 1.02ab	20.94 ± 1.65a	115.63 ± 15.13a	29.69 ± 2.87ab	59.63 ± 9.53ab	28.06 ± 2.52b	33.25 ± 1.84a	133.00 ± 7.38a	38.44 ± 1.09a
OMF	426.31 ± 68.00a	133.31 ± 5.58a	10.88 ± 1.02a	20.81 ± 2.26a	114.06 ± 8.98a	30.31 ± 2.87a	64.94 ± 11.33a	30.50 ± 2.50a	32.94 ± 2.49a	131.75 ± 9.96a	37.88 ± 1.71ab

Different letters (a, b) on the same column indicate values that are significantly different (P < 0.05) based on one-way ANOVA and LSD post-hoc test.

**Figure 1:**
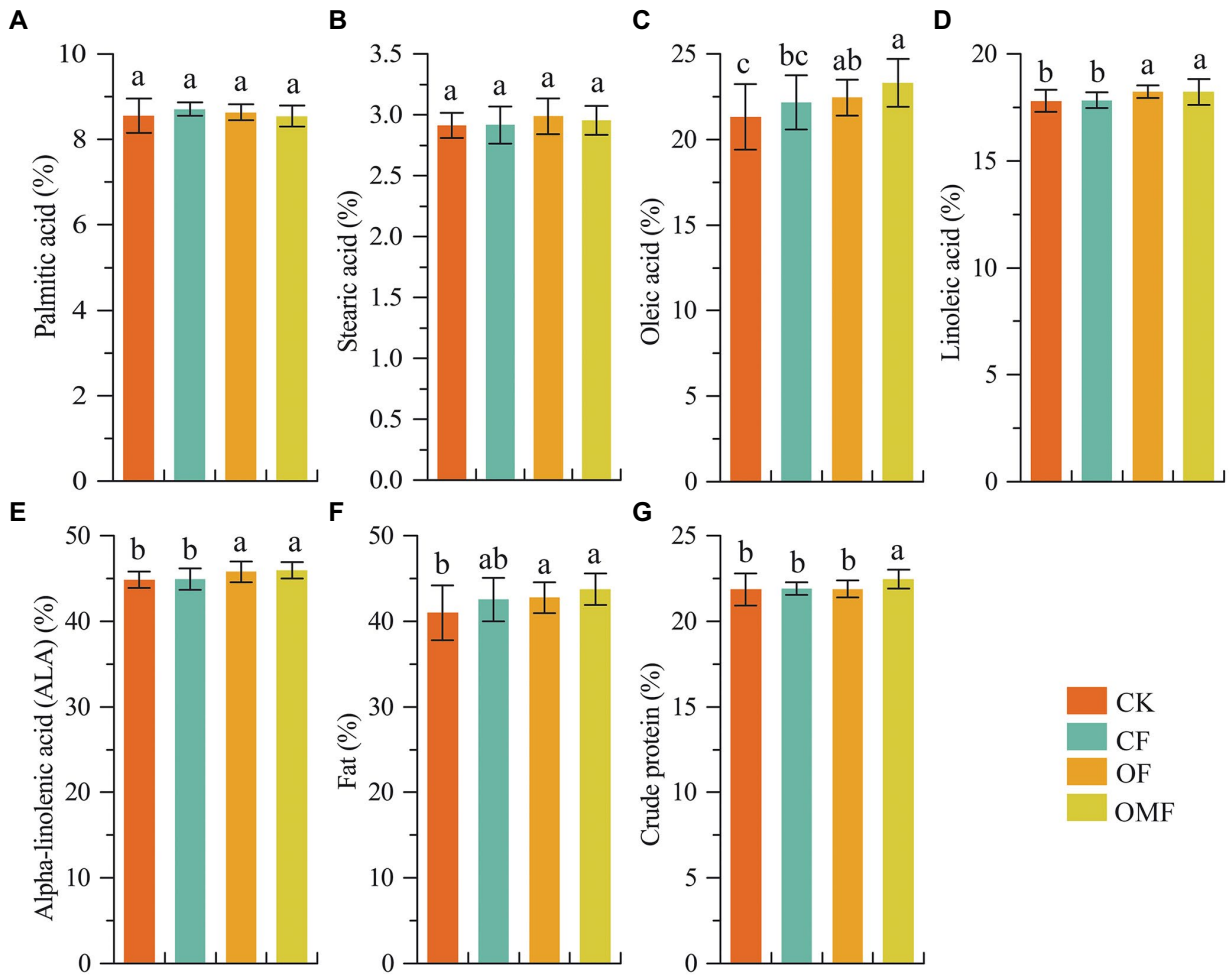
Perilla frutescens quality index under different fertilization treatments. Based on one-way ANOVA and LSD post hoc test, different letters (a,b) indicated significant differences among fertilization treatment groups (*P* < 0.05).

Analysis of *P. frutescens* seed quality showed that the contents of oleic acid, linoleic acid, α-linolenic acid, and fat in the OF and OMF groups were significantly higher than those in the CK and *CF* groups. Additionally, the OMF group had the highest contents of oleic acid, α-linolenic acid, and fat (Figure 1). The crude protein content of the OMF group was significantly higher than that of the other treatment groups (*p* < 0.05). This indicated that the application of OFs, especially OMF, helped improve *P. frutescens* quality.

### Effects of fertilization treatments on soil microbial community composition

**Figure 2:**
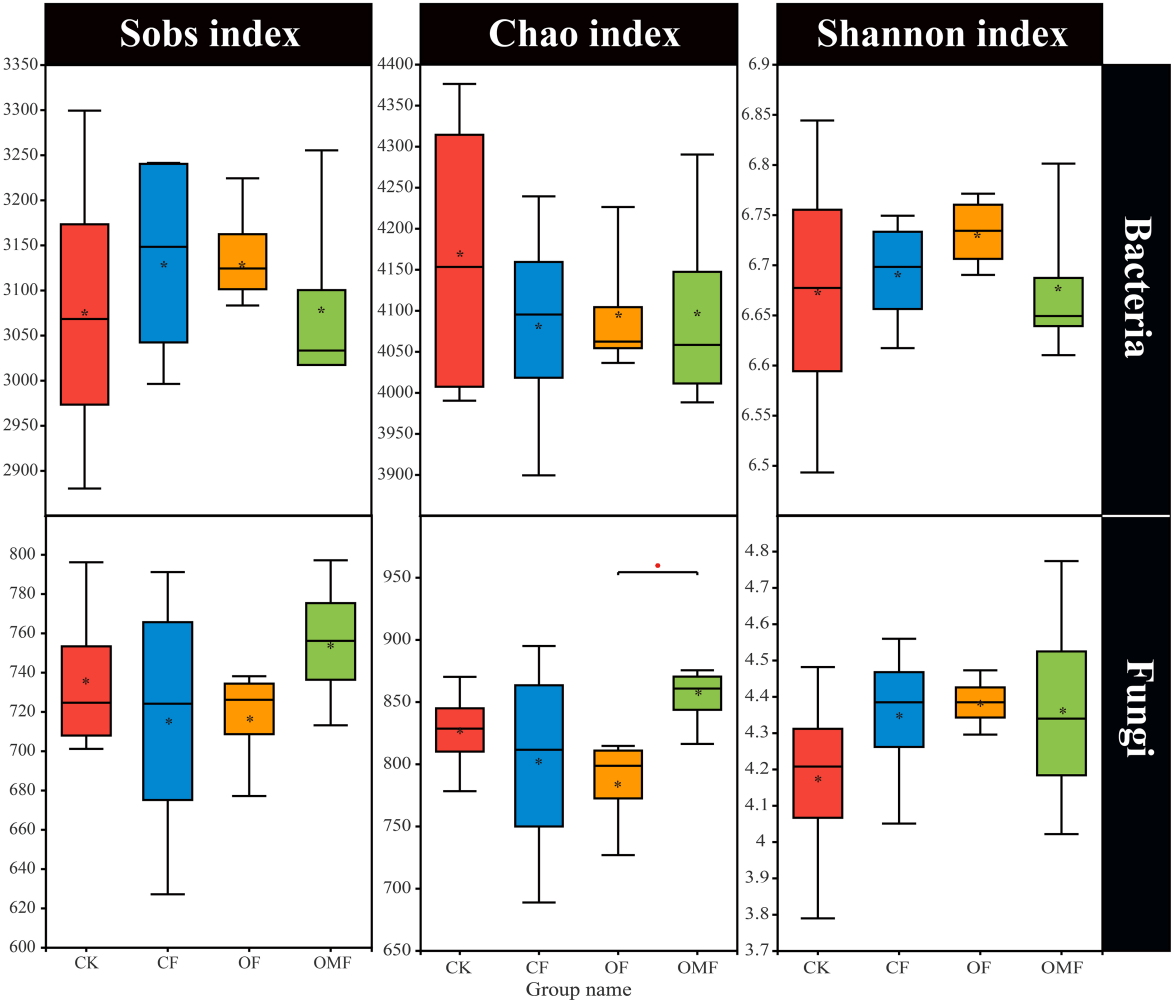
Alpha diversity index of soil microbial community in different treatment groups. *indicates a significant difference at the level of 0.01 < *p* ≤ 0.05 (Student *t* test).

**Figure 3:**
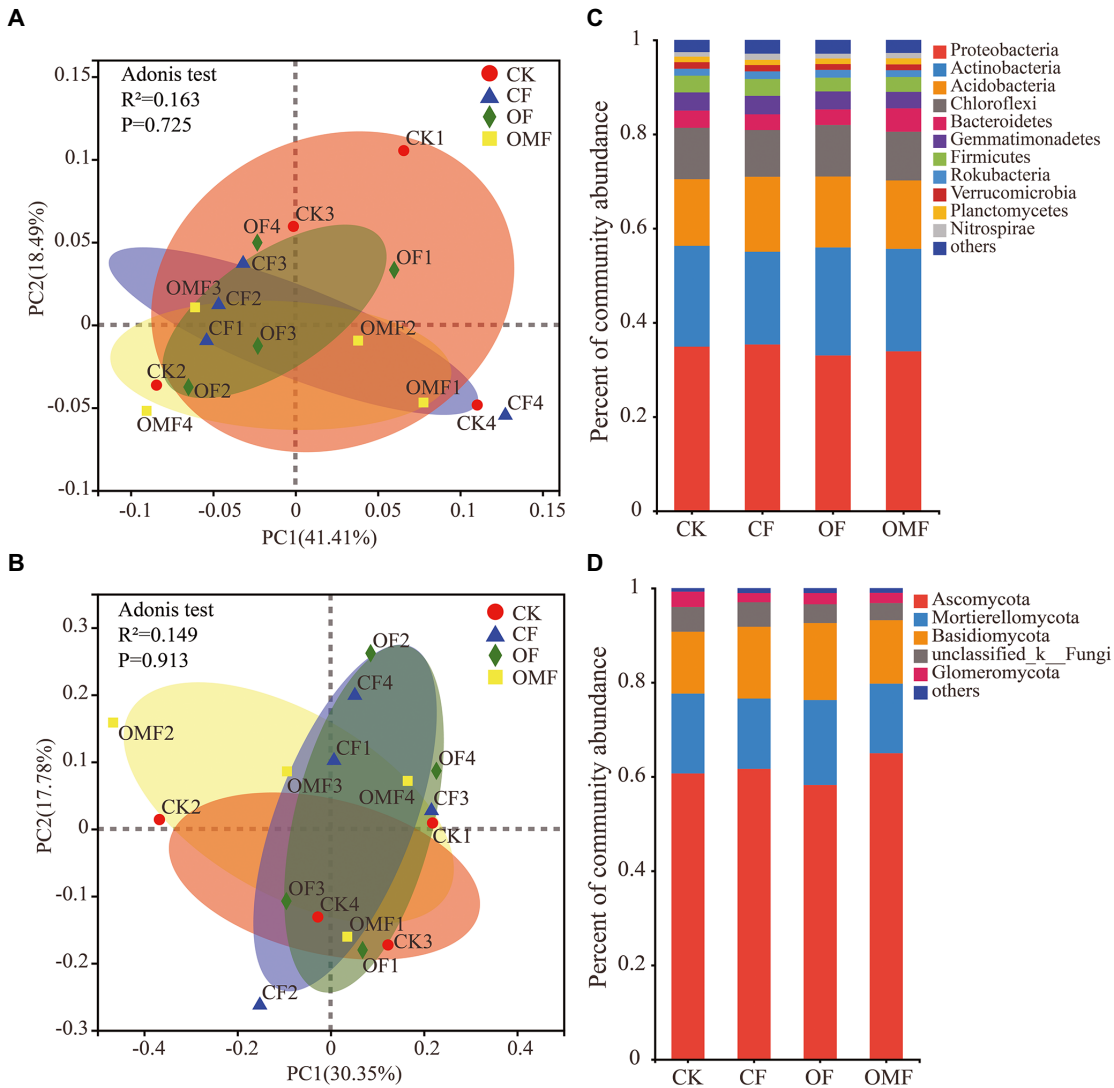
Principal coordinates analysis of the Weighted-Unifrac distance matrix for bacteria **(A)**, and fungi **(B)**, in different fertilizer treatment groups. Bar plots of relative abundance of bacterial phyla **(C)**, and fungal phyla **(D)**, in different fertilization groups.

Alpha diversity analysis showed that different fertilization treatments did not significantly affect soil bacterial community richness and diversity, whereas OMF significantly increased soil fungal community richness (Figure 2). PCoA showed that there was no clear distinction between samples from different treatment groups on the PC1 and PC2 axes (Figures 3A,B); this indicated that there was no significant difference in soil bacterial community and fungal community composition among different fertilizer treatment groups in the short term.

Fertilization management did not cause significant changes in phylum-level bacterial composition (*p* > 0.05, ANOVA analysis; Figure 3C). Proteobacteria, Actinobacteria, Acidobacteria, and Chloroflexi were the main dominant bacterial phyla in each fertilization treatment group. Fertilization management also did not significantly affect the fungal community composition at the phylum level (*p* > 0.05, ANOVA; Figure 3D). Ascomycota, Mortierellomycota, Basidiomycota, and Glomeromycota were the dominant fungal phyla in each treatment group. The taxonomic analysis of the dominant bacterial genera with relative abundance >1% showed that different short-term fertilization treatments had no obvious effect on most dominant bacterial genera (Supplementary Table S1). However, the relative abundances of *Bacillus* and *Candidatus_Solibacter* significantly increased after fertilization, especially in the OF and OMF groups; the relative abundance of *Bryobacter* significantly decreased in the OF and OMF groups. The relative abundances of *Clonostachys* and *Gonytrichum* in the OMF group were significantly lower than those in the other groups, and the relative abundance of *Penicillium* in the OF group was significantly lower than that in the other treatment groups. In addition, there were no significant differences in other dominant fungal genera among the treatment groups (Supplementary Table S2).

**Figure 4:**
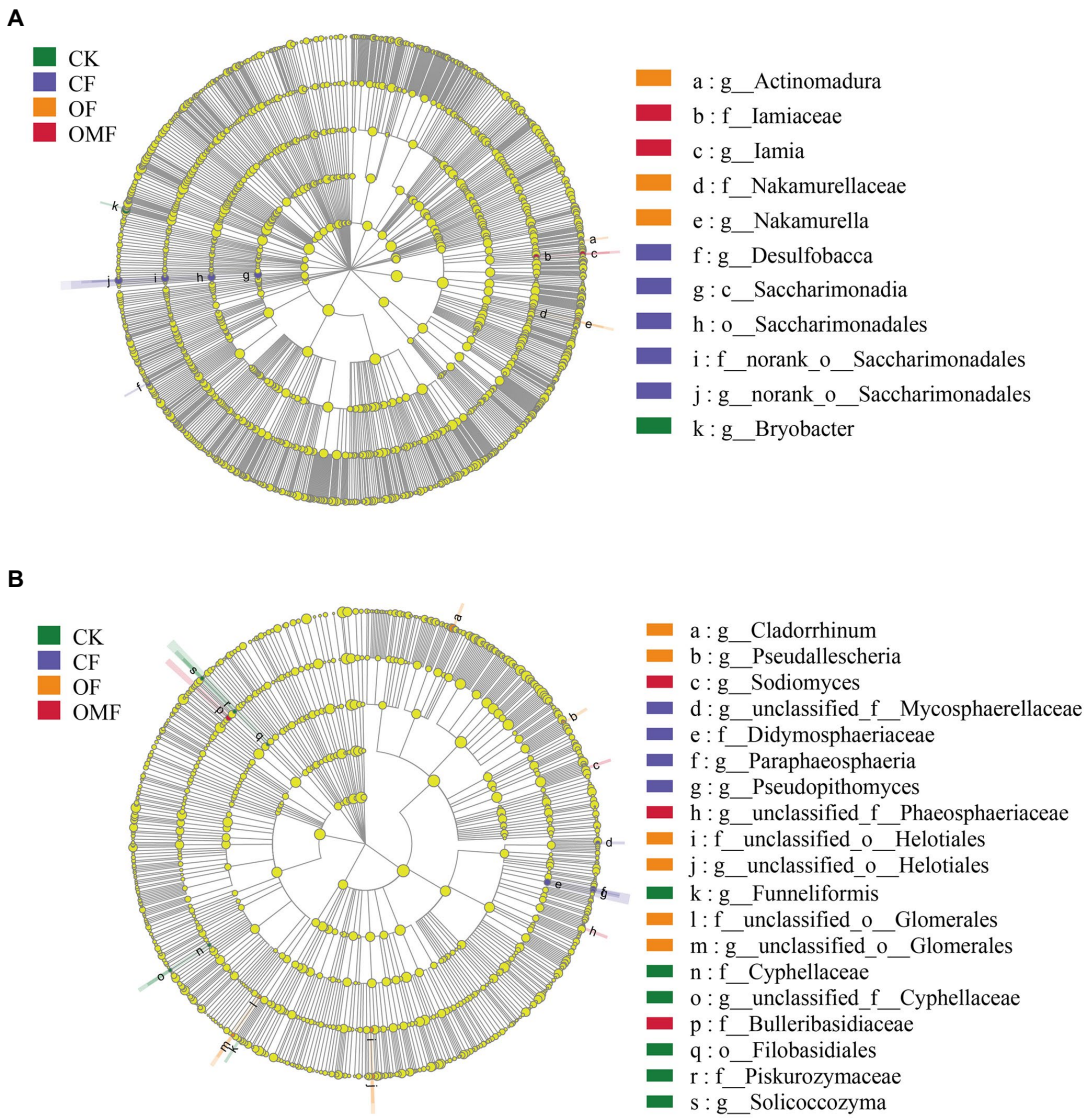
Effects of different fertilization treatments on the relative abundance of soil bacterial **(A)**, and fungal **(B)**, lineages. The linear discriminant analysis (LDA) effect size analysis was performed to identify the indicator taxa representing each group, and the values were significant (*p* < 0.05) when the LDA score was greater than 4. There are five rings in the cladogram, that represent the phylum, class, order, family, and genus from inside to outside, respectively. The different color nodes (except yellow, which indicates no significant changes) on the ring represent significant changes in taxonomic composition due to the treatments. Abbreviations for classification levels: P, phylum; C class; O, order; F, family; G, genus.

The LEFSe results showed that *Bryobacter* was significantly enriched in the CK group; the nitrogen-fixing bacteria *Saccharimonadia* and iron-reducing bacteria *Desulfobacca* were significantly enriched in the *CF* group; *Actinomadura*, *Nakamurellaceae* and *Nakamurella* were significantly enriched in the OF group; and *Iamia* and *Iamiaceae* was significantly enriched in the OMF group (Figure 4A). The LEFSe analysis of the fungal community showed (Figure 4B) that Cyphellaceae, Piskurozymaceae, Filobasidiales, *Solicoccozyma*, and *Funneliformis* were mainly enriched in the CK group, and Mycosphaerellaceae, Didymosphaeriaceae, *Paraphaeosphaeria*, and *Pseudopithomyces* were mainly enriched in the *CF* group. Helotiales, Glomerales, *Cladorrhinum*, and *Pseudallescheria* in the OF group, and Phaeosphaeriaceae, Bulleribasidiaceae, and *Sodiomyces* were mainly enriched in the OMF group.

### Co-occurrence network analysis of soil microbial communities under different fertilization management techniques

**Figure 5:**
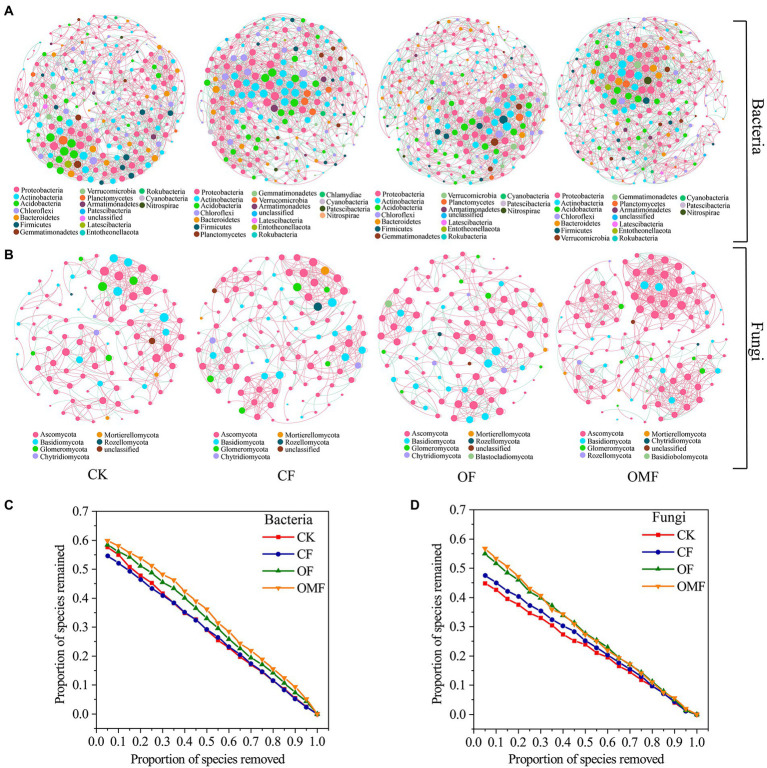
Co-occurrence networks of the soil microbial communities at the genus level in different fertilization treatment groups **(A,B)**; and the robustness of bacterial network **(C)**, and fungal network **(D)**. The node size is proportional to the taxon abundance, and the nodes represent bacterial or fungi taxa at the genus level (genera with relative abundances greater than 0.1%). The node colors represent different bacterial and fungal phyla. The edges are colored according to interaction types; positive correlations are labeled in pink and negative correlations are labeled in green.

Analysis of the topological indicators of the bacterial community co-occurrence network revealed that the OMF group had the highest increase in the number of network edges and average degree (Table 4). The OF group had the highest modularity index, followed by the OMF group. This finding indicated that the application of OF and OMF could enhance the association of soil bacterial communities because the degree of modularity was higher. The robustness analysis results showed that the robustness of OF and OMF treatment groups were higher than that of CK and OF groups, and OMF groups was highest (Figure 5C).

**Table 4:** Topological indices of each co-occurrence network in Figure 5.

	Bacteria				Fungi			
	CK	*CF*	OF	OMF	CK	*CF*	OF	OMF
No. of edges^1^	1,629	1,915	1,833	1,985	411	473	420	659
Modularity^2^	3.702	5.209	7.045	5.807	1.154	1.190	1.584	1.808
Graph density^3^	0.065	0.077	0.070	0.076	0.075	0.080	0.071	0.097
Average degree^4^	14.480	17.175	15.939	17.336	7.829	8.679	7.706	11.265
Average path length^5^	4.508	4.306	4.274	4.390	7.941	7.498	5.829	3.252
Average clustering coefficient^6^	0.660	0.666	0.648	0.659	0.752	0.760	0.703	0.778

1 Number of connections/correlations obtained by Gephi software.

2 Capability of the nodes to form highly connected communities, that is, a structure with high density of between nodes connections.

3 Measure network integrity. A complete graph with all possible edges, that is, any two nodes with edge connections, has a density of 1.

4 Average number of connections per node in the network, that is, the node connectivity.

5 Average network distance between all pair of nodes or the average length off all edges in the network.

6 How nodes are embedded in their neighborhood and the degree to which they tend to cluster together.

Analysis of the network topology indicators of the fungal community showed that, compared with other treatment groups, the OMF group significantly increased the total number of edges in the network, number of positive correlation edges, number of nodes, graph density, and average degree (Table 4). The OMF group had the highest modularity index; this demonstrated that OMF can improve the association of fungal communities, and OMF can make the fungal community structure more modular and more stable. The robustness analysis results showed that the robustness of OMF and *CF* groups had little difference, but was higher than that of CK and OF groups (Figure 5D).

Analysis of the scale proportions of the top three modules in each group revealed that the proportion of the top three modules of bacterial and fungal communities was greatest in the OF group followed by the OMF group (Supplementary Figure S2). This indicated that the bacterial communities and the fungal communities of the OF and OMF groups were more closely related.

The main nodes in the bacterial community network of each treatment group belonged to Proteobacteria, Actinobacteria, Acidobacteria, Chlorobacteria, Bacteroidetes, and Firmicutes; the main nodes in the fungal community network belonged to Ascomycota, Basidiomycota, and Glomeromycota. This finding indicated that these bacterial and fungal phyla were keystone microbiota in all treatment groups (Figure 5). According to the analysis of the betweenness centrality values (Supplementary Table S3), the top 10 genus-level species were different in each treatment group, which indicated that the genus-level microbiota that played a key role in the co-occurrence network differed among treatment groups.

### Effects of different fertilizer treatments on the ecological function of soil microbial communities

**Figure 6:**
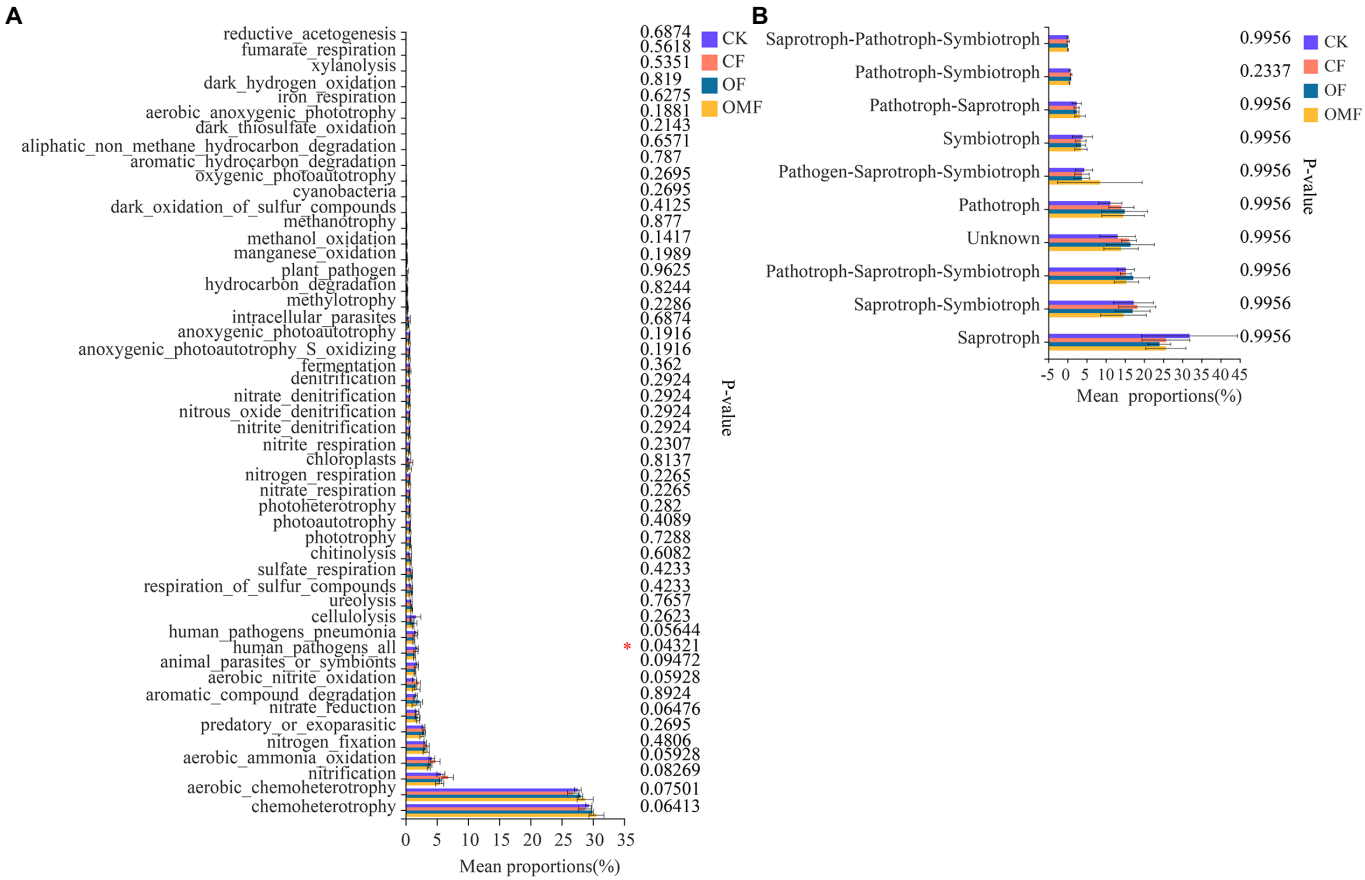
Functional analysis of soil microbial community in different treatment groups. FAPROTAX function analysis of bacterial communities **(A)**, and FUNGuild function analysis of fungal communities **(B)**. The abscissa indicates the function name, the ordinate indicates the percentage value of a function abundance of the sample, and different colors indicate different groups. Based on Kruskal–Wallis H test, the rightmost values represent *p* values, where *indicates 0.01 < *p* ≤ 0.05.

According to FAPROTAX functional analysis, chemoheterotrophy, aerobic chemoheterotrophy, nitrification, aerobic ammonia oxidation, and nitrogen fixation were the top five bacterial community functions in each treatment group (Figure 6A). Among the top 50 functions, only human pathogens all significantly differed among different treatments, and the OF and OMF treatments reduced the proportion of human pathogenic bacteria in soil compared with CK and *CF* treatments. There were no significant differences in other bacterial community functions among different treatment groups.

FUNGuild functional analysis indicated that Saprotroph, Saprotroph–Symbiotroph, Pathotroph–Saprotroph–Symbiotroph, Pathotroph, and Symbiotroph were the top five trophic modes of the fungal community (Figure 6B). Analysis of the relative abundance of arbuscular mycorrhizal fungi (AMF) in symbiotic trophic fungi showed that the AMF abundance was significantly reduced in the *CF* group (Supplementary Figure S3A); this indicated that CFs inhibited AMF growth. F_unclassified_o_Paraglomerales, Glomeraceae, Diversisporales_fam_Incertae_sedis, f_unclassified_o_GS24, and Paraglomeraceae were the dominant families in AMF, but there were significant differences in their relative abundances among different fertilization treatments groups (Supplementary Figure S3B).

### Effects of soil environmental factors on microbial community composition and diversity, and *P. frutescens* yield and quality

**Figure 7:**
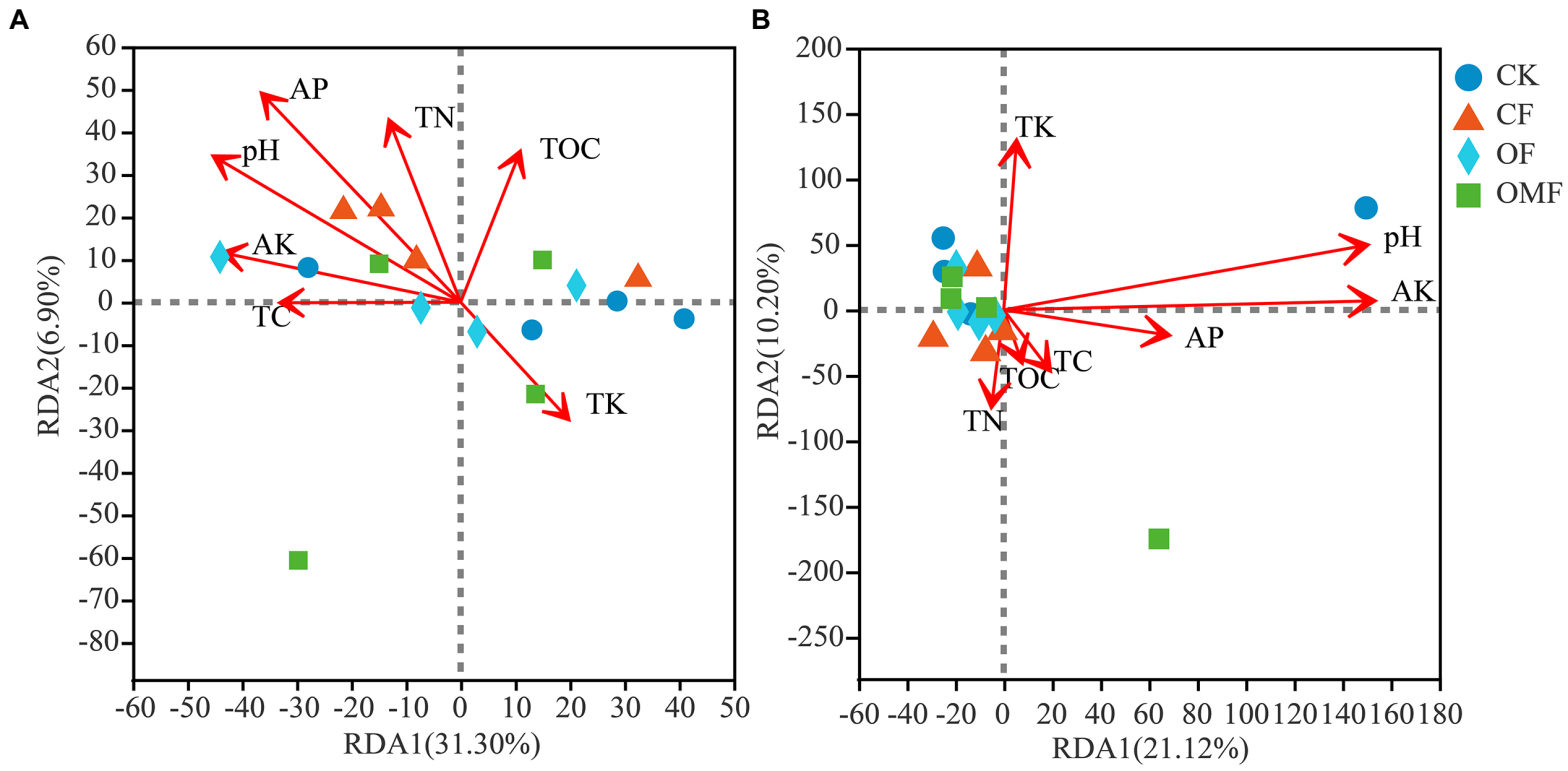
Distance-based redundancy analysis (RDA) among bacterial **(A)**, or fungal **(B)**, communities and environmental factors.

RDA indicated that the selected environmental factors explained 38.20% of the total change in bacterial communities (Figure 7A) and 31.32% of total changes in fungal communities (Figure 7B). The results of RDA showed that pH (ANOVA, *p* = 0.021) and AP (ANOVA, *p* = 0.024) were the main factors affecting the soil bacterial community composition, and pH (ANOVA, *p* = 0.034) and AK (ANOVA, *p* = 0.011) were the main factors affecting the soil fungal community composition (Figure 7).

**Figure 8:**
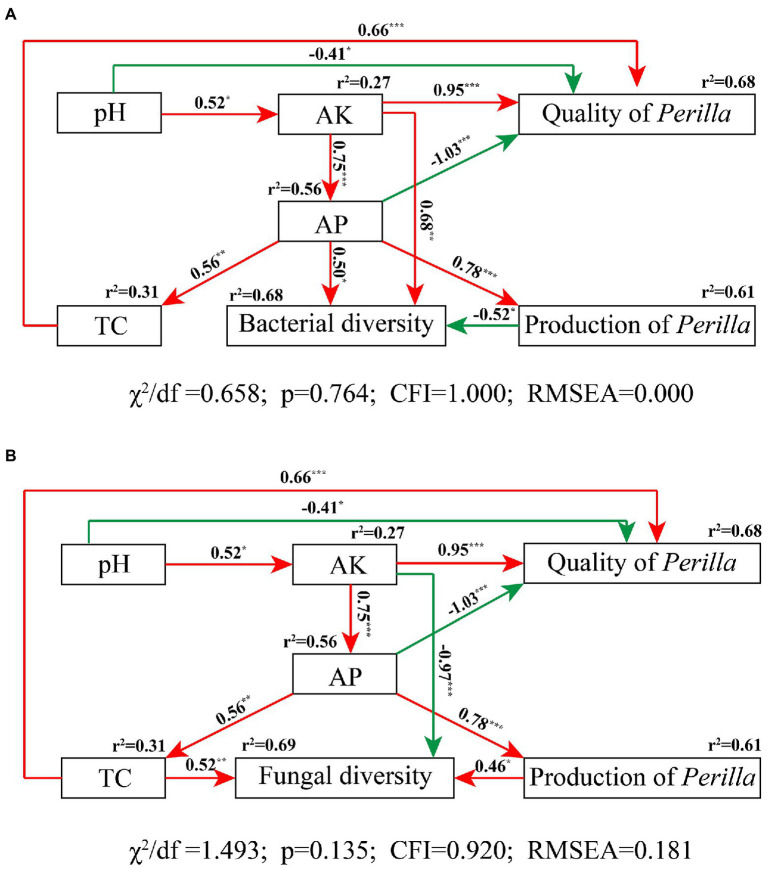
Structural equation modeling results describing the relationship among soil nutrients, microbial diversity, and *P. frutescens* yield and quality. The relationship among soil pH value, total soil carbon content (TC), available potassium (AK), available phosphorus (AP), *P. frutescens* production, and *P. frutescens* quality with bacterial diversity **(A)**, and fungal diversity **(B)**. Red lines: positive correlation; green lines: negative correlation. The numbers above the arrows indicate correlation strength. *r*^2^ values indicate the proportion of variance explained for each variable. *χ*^2^, Chi-square; df, degrees of freedom; p, probability level; RMSEA, goodness-of-fit statistics for each model. Significance levels of each predictor are shown as *for *p* < 0.05, **for *p* < 0.01, and ***for *p* < 0.001.

The SEM fit the measured data well (bacteria, *χ*^2^/df = 0.658, *p* = 0.764, CFI = 1.000, RMSEA = 0.000; fungi, *χ*^2^/df = 1.493, *p* = 0.135, CFI = 0.920, RMSEA = 0.181); this indicated high consistency between the hypothesized model and the observed data (Figure 8). The SEM showed that soil physicochemical properties accounted for 68% of *P. frutescens* quality, among which AK and TC were significantly positively correlated with *P. frutescens* quality, and pH and AP were significantly negatively correlated with *P. frutescens* quality (Figure 8). AK and AP were significantly positively correlated with bacterial diversity, and *P. frutescens* yield was significantly negatively correlated with bacterial diversity; these factors accounted for 68% of bacterial diversity (Figure 8A). AP was significantly positively correlated with and explained 61% of *P. frutescens* yield. TC and *P. frutescens* yield were directly and significantly positively correlated with fungal community diversity, whereas AK was directly and significantly negatively correlated with fungal community diversity (Figure 8B).

## Discussion

### Effects of organomineral fertilizer on soil properties and *P. frutescens* yield and quality

Compared with the CK group, OMF application significantly increased the TC and TK contents of the soil (Table 2). The experiment proved that although short-term application of OMF could not significantly improve most of the soil physicochemical properties, it could guarantee the same effect as the same amount of inorganic fertilizer. Numerical analysis showed that OF especially OMF application had a trend of improving soil fertility compared with no fertilization and *CF* application, which was consistent with previous continuous fertilization results (Zhao et al., 2016; Du et al., 2022).

The yield test results of *P. frutescens* at harvest showed that the number of panicles per plant and length of the main panicle were significantly greater in the OMF group compared with the other treatment groups (Table 3); this indicated that OMF increased *P. frutescens* yield to a certain extent. The quality inspection results of *P. frutescens* showed that OF and OMF treatments significantly increased the contents of unsaturated fatty acids, total fat, and total protein in *P. frutescens*, and the OMF treatment values were higher (Figure 1). These results were similar to those of a previous study that investigated the effect of OMF on *Purslane* growth, which also showed that OMF treatment increased unsaturated fatty acid content (Yang et al., 2020). The SEM results also demonstrated that soil AK content was significantly positively correlated with *P. frutescens* quality (Figure 8). Therefore, OMF application was beneficial for improving *P. frutescens* quality.

### Effects of different fertilization treatments on soil microbial communities

Microorganisms are the driver of soil fertility changes; they can directly indicate soil quality and play an important role in plant growth and crop yield (Fan et al., 2020; Finkel et al., 2020). In this study, short-term fertilization treatments did not significantly affect alpha diversity (Figure 2). Similar studies also showed that soil microbial alpha diversity was stable and not easily affected by agricultural management practices (Coller et al., 2019; Gui et al., 2021; Kang et al., 2022). How fertilization management affects soil microbial diversity depends on soil properties (Mendes et al., 2015), such as soil pH, which is generally considered a decisive factor underlying microbial diversity (Bissett et al., 2011).

Actinobacteria and Firmicutes are generally considered to be beneficial microorganisms for plants (Yang et al., 2017). Actinobacteria can control plant bacterial diseases by producing various antibiotics, secreting cell wall-degrading enzymes, and inducing host resistance (Conn et al., 2008; Chater et al., 2010; Liu et al., 2012). LEFSe analysis showed that the treatment of OF and OMF promoted the enrichment of some bacteria belong to Actinobacteria, including *Actinomadura*, *Nakamurellaceae*, *Nakamurella*, *Iamiaceae*, and *Iamia* (Figure 4). *Actinomadura* can produce several antibiotics that inhibit the growth of soil pathogens and reduce the occurrence of crop diseases and insect pests (Li et al., 2022). *Bacillus* and *Candidatus_Solibacter* were also significantly increased in OF and OMF groups (Supplementary Table S1). *Bacillus* is commonly formulated as biocontrol agents because they secrete antibiotics or antimicrobial proteins (Ahimou et al., 2000; Weller et al., 2002; Moyne et al., 2004), and improve soil fertility by increasing soil mineral nutrient availability (Chen et al., 2016). Additionally, *Candidatus_Solibacter* is a bacterium that decomposes organic matter (Rime et al., 2015).

LEFSe analysis showed that the main enriched fungal species (e.g., Glomerales, *Cladorrhinum*, and *Pseudallescheria*) in the OF groups belonged to the phyla Ascomycota and Glomeromycota (Figure 4B). Among them, *Pseudallescheria* is a biocontrol fungus; it is an important natural enemy of some plant parasitic nematodes that can parasitize eggs and infect larvae and females, and can significantly reduce the damage of plant nematode diseases such as those caused by root-knot, cyst, and stem nematodes of various crops (Wang et al., 1997; Ko et al., 2010; Zhu et al., 2020). *Cladorrhinum* is an effective biocontrol fungus for controlling the soil-borne *Rhizoctonia solani* pathogen (Liu, H. et al., 2019). Ascomycota is a key driver of the degradation of organic residues in soil (Richardson, 2009; Ma et al., 2013); therefore, the Ascomycota abundance may increase with increasing organic matter content (Du et al., 2022). Glomeromycota can undergo symbiosis with terrestrial plants to form arbuscular mycorrhizae, and this symbiosis can help plants absorb inorganic salts in soil, especially phosphorus (Smith and Read, 2008; Calaca and Bustamante, 2022). Our results showed that, OF treatments promoted increase in the number of beneficial fungi in the karst soil.

In conclusion, short-term fertilization treatments affected soil microbial communities, and OF and OMF had advantages over *CF.* However, compared with previous long-term experimental results, some differences in this study were not significant. Therefore, extending the fertilization period and intensifying fertilization may produce significant fertilization effects (Kox et al., 2020).

### OMF treatment increased connectivity and structural stability of soil microbial communities

There is a complex association network among soil microbial communities, and they do not exist alone. When soil microbial community composition changes because of fertilization management, the microbial co-occurrence network also changes (Kang et al., 2022). The OF and OMF groups had higher modularity indices, which indicated that OF and OMF improved the soil microbial community connectivity and made the community connected more closely (Figure 5; Supplementary Figure S2; Table 4). This result is generally consistent with those of other studies on OF application (Ling et al., 2016; Wang et al., 2017; Liu et al., 2020; Kang et al., 2022).

Complex networks with higher connectivity are more tolerant of environmental disturbances than simple networks with lower connectivity (Santolini and Barabási, 2018). In this study, the network connectivity of both bacterial and fungal communities was highest in the OMF group (Table 4); this indicated that OMF treatment resulted in higher anti-interference ability of soil microorganisms. As the core components of soil organic matter degradation, bacteria and fungi usually form different functional groups and change the interaction between their ecological networks because of the decomposition or utilization of organic and inorganic nutrients (Wang et al., 2017; Dai et al., 2018; Samaddar et al., 2019), and they tend to maintain a complex network structure (Kang et al., 2022). The robustness analysis showed that the application of OF, especially OMF, could improve the stability of bacterial and fungal network structures (Figures 5C,D).

Keystone microbial groups play an important role in maintaining ecosystem homeostasis (Banerjee et al., 2018; Fan et al., 2020). According to the degree values of the network nodes, it was found that the key bacterial and fungal phyla in the nodes were not significantly different among the treatment groups (Figure 5); this was consistent with the composition and distribution of dominant species in the community (Figures 3C,D). At the phylum level, microorganisms had strong stability and were not easily affected by fertilization management. The genera with the highest betweenness centrality scores are generally considered keystone taxa (González et al., 2010; Vick-Majors et al., 2014). In this study, the keystone genera differed among fertilization treatment groups. The keystone genus of the bacterial community in the OMF group was *Pseudomonas*, and the keystone genus of the fungal community was *Metarhizium* (Supplementary Table S3), both of which are recognized as biocontrol microorganisms. *Pseudomonas* can adsorb heavy metals in soil and promote plant growth (Costa-Gutierrez et al., 2020; Ghorbanzadeh et al., 2020; Wu et al., 2022), whereas *Metarhizium* can kill plant pests and mitigate plant diseases (Riguetti Zanardo Botelho et al., 2019; Gebremariam et al., 2021; González-Pérez et al., 2022). In conclusion, this study demonstrated that the application of organic fertilizer, especially OMF, can not only enhance the connectivity, cohesiveness and stability of microbial community network structure, but also increase the abundance of beneficial microorganisms.

### Relationship among soil physicochemical properties, soil microbial community, and *P. frutescens* yield and quality

The RDA results showed that soil pH was the most important factor affecting bacterial and fungal communities (Figure 7). This results was consistent with the previous study (Cho et al., 2016; Gu et al., 2019). Changes in soil pH can alter soil structure, fertility, and vegetation communities, thereby directly or indirectly affecting soil microbial community composition (Lauber et al., 2009; Qi et al., 2018). In addition, in this study, AP was also the main factor affecting bacterial communities, and AK was the main factor affecting fungal communities (Figure 7). Fertilizers may be absorbed and used by plants after entering the soil, or they may remain in the soil, leading to changes in the composition of bacterial communities (Sun et al., 2016) and fungal communities (Zhou et al., 2016). Therefore, different fertilization treatments affected soil microbial community composition by mediating the effects of soil physicochemical properties, especially pH, and AP and AK.

SEM results showed that both soil TC and AK content was positively correlated to the quality (linoleic acid content) of *P. frutescens* (Figure 8), indicated that improve soil carbon and available potassium content could improve the quality of *P. frutescens*. There was a direct and significant positive correlation between *P. frutescens* yield and AP (Figure 8); this indicated that *P. frutescens* yield was mainly affected by the AP content in soil, and increasing the AP content can improve *P. frutescens* yield. Soil bacterial diversity was significantly positively correlated with soil AK (Figure 8A), but fungal diversity was significantly negatively correlated with soil AK (Figure 8B). Some previous studies showed that soil bacteria and fungi exhibited different patterns in response to fertilization treatments (Álvarez-Martín et al., 2016; Ai et al., 2018), and our study also supported this conclusion. Fungi are generally considered more closely related to plants and they are able to provide nutrients to plants in a symbiotic relationship (Chen et al., 2017), whereas bacteria are more affected by soil properties and environmental factors (Singh et al., 2008; Delgado-Baquerizo et al., 2016, 2018); our study obtained similar results (Figures 7, 8). In this study, we found that soil bacterial diversity was significantly negatively correlated with *P. frutescens* yield, and fungal diversity was significantly positively correlated with *P. frutescens* yield. This result may be opposite to many previous long-term fertilization studies. Because, in this study, short-term application of OF and OMF significantly increased *P. frutescens* yield (biomass per plant), but there was no significant change in bacterial diversity (Shannon index), and the value of OMF group even showed a downward trend (Figure 2). The application of organic fertilizer may stimulate the rapid growth of some dominant bacteria and beneficial bacteria in soil in a short time, while the abundance of some oligotrophic microorganisms that are not adapted to the existence of organic fertilizer will decline, resulting in the decrease of bacterial diversity. Through the correlation analysis among soil physicochemical properties, microbial communities and *P. frutescens* agronomic efficiency, it can be concluded that the application of OF or OMFs can improve the physicochemical properties of soil, especially the contents of total carbon, available potassium and available phosphorus, which can promote the quality and yield of *P. frutescens*.

## Conclusion

The results of this study demonstrated that, under short-term fertilization management, OMF increased the total carbon and total potassium contents of soil. OF, especially OMF, improved measures of *P. frutescens* yield and quality, including the number of panicles per plant, length of the main panicle, and contents of unsaturated fatty acids such as α-linolenic acid, total fat, and total protein, while significantly increasing the number of beneficial microbial communities in the soil. The co-occurrence network analysis also revealed that OF and OMF improved the connectivity and stability of soil microbial communities. In conclusion, application of OF, especially OMF, is a good strategy to shape the composition of beneficial bacterial communities in the soil, and to improve soil fertility and crop yield and quality in karst areas.

## Data availability statement

The datasets presented in this study can be found in online repositories. The names of the repository/repositories and accession number(s) can be found at: https://www.ncbi.nlm.nih.gov/, PRJNA836163; https://www.ncbi.nlm.nih.gov/, PRJNA836186.

## Author contributions

BL and XL designed the study and modified the manuscript. YL and QS did the experimental work, carried out the data analysis, and wrote the manuscript. XA and YX contributed to the data analysis and original draft writing. All authors contributed to the article and approved the submitted version.

## Funding

This work was supported by the Strategic Priority Research Program of the Chinese Academy of Science (XDA23060102), the Project on Social Development by Department of Science and Technology of Guizhou Province (SY-[2014]3041), the talents of Guizhou Science and Technology Cooperation Platform (2016-5648) and the Opening Fund of the State Key Laboratory of Environmental Geochemistry (SKLEG2021XXX).

## Conflict of interest

The authors declare that the research was conducted in the absence of any commercial or financial relationships that could be construed as a potential conflict of interest.

## Publisher’s note

All claims expressed in this article are solely those of the authors and do not necessarily represent those of their affiliated organizations, or those of the publisher, the editors and the reviewers. Any product that may be evaluated in this article, or claim that may be made by its manufacturer, is not guaranteed or endorsed by the publisher.

## Footnotes

^1^ http://vdb3.soil.csdb.cn/

^2^ http://www.usadellab.org/cms/?page=trimmomatic

^3^ https://ccb.jhu.edu/software/FLASH/

^4^ http://drive5.com/uparse/

^5^ http://www.arb-silva.de

^6^ http://unite.ut.ee

^7^ https://gephi.org/
