# Supplementary material for: Organomineral fertilizer application enhances Perilla frutescens nutritional quality and rhizosphere microbial community stability in karst mountain soils
Source: Front Microbiol. 2022 Nov 24;13:1058067. doi: 10.3389/fmicb.2022.1058067 (PMC9730529; doi:10.3389/fmicb.2022.1058067)

**Supporting Information：**

**Organomineral fertilizer application enhances *Perilla*** ***frutescens* nutritional quality and rhizosphere microbial community stability in karst mountain soils**

Ying Li^1,3†^, Qi Shen^2†^, Xiaochi An^3^, Yuanhuan Xie^1^, Xiuming Liu^1*^, Bin Lian^3*^

1. State Key Laboratory of Environmental Geochemistry, Institute of Geochemistry, Chinese Academy of Sciences, 99 Lincheng Road West, Guiyang 550081, China

2. Institute of Medical Plant Physiology and Ecology, School of Pharmaceutical Sciences, Guangzhou University of Chinese Medicine, Guangzhou 510006, China

3. College of Life Sciences, College of Marine Science and Engineering, Nanjing Normal University, Wen Yuan Road, Nanjing 210023, China

*Corresponding author

Bin Lian

bin2368@vip.163.com

Xiuming Liu

liuxiuming@vip.skleg.cn

^†^Equal contribution and first authorship: Ying Li^†^ and Qi Shen^†^ have contributed equally to this work and share first authorship.

**Table S1** Distribution of dominant bacteria in different fertilization treatment groups based on genus level classification.

| **Genus** | **Relative abundance (%)** | | | |
| --- | --- | --- | --- | --- |
|  | **CK** | **CF** | **OF** | **OMF** |
| *Bradyrhizobium* | 1.83±0.29a | 1.88±0.21a | 1.72±0.21a | 1.93±0.41a |
| *Flavobacterium* | 0.75±0.50a | 0.63±0.31a | 0.56±0.19a | 1.75±1.49a |
| *Sphingomonas* | 1.71±0.09a | 1.68±0.26a | 1.58±0.18a | 1.71±0.36a |
| *Bacillus* | 1.18±0.23b | 1.33±0.16b | 1.61±0.22a | 1.46±0.34a |
| *Gemmatimonas* | 1.40±0.21a | 1.30±0.08a | 1.31±0.35a | 1.24±0.19a |
| *Gaiella* | 1.11±0.10a | 0.97±0.28a | 1.26±0.03a | 1.20±0.22a |
| *Haliangium* | 1.32±0.22a | 1.28±0.13a | 1.24±0.05a | 1.19±0.12a |
| *Mycobacterium* | 1.14±0.18a | 1.19±0.16a | 1.10±0.09a | 1.18±0.14a |
| *Candidatus_Solibacter* | 1.03±0.27b | 1.16±0.18b | 1.64±0.55a | 1.50±0.23a |
| *Nitrospira* | 0.92±0.24a | 1.25±0.12a | 0.98±0.13a | 1.11±0.43a |
| *Bryobacter* | 1.39±0.39a | 1.32±0.07a | 0.97±0.18b | 1.06±0.08ab |
| *RB41* | 0.85±0.26a | 1.15±0.37a | 1.03±0.34a | 1.05±0.34a |
| *Nocardioides* | 1.01±0.19a | 0.72±0.13a | 1.19±0.40a | 1.04±0.46a |
| *Ellin6067* | 1.16±0.26a | 0.98±0.21a | 0.94±0.12a | 0.93±0.16a |

**Table S2** Distribution of dominant fungi in different fertilization treatment groups based on genus level classification.

| **Genus** | **Relative abundance (%)** | | | |
| --- | --- | --- | --- | --- |
|  | **CK** | **CF** | **OF** | **OMF** |
| *Mortierella* | 16.18±5.13a | 13.94±4.76a | 15.46±3.84a | 13.83±5.73a |
| *Acremonium* | 4.34±2.22a | 3.77±1.89a | 3.74±2.06a | 8.46±11.04a |
| *Saitozyma* | 7.39±2.00a | 6.21±2.15a | 8.59±4.03a | 7.21±2.57a |
| *Plectosphaerella* | 2.29±0.75a | 2.84±1.04a | 2.96±0.54a | 6.68±5.87a |
| *Fusarium* | 4.11±1.20a | 4.01±1.13a | 4.03±0.59a | 4.06±0.92a |
| *Sarocladium* | 0.01±0.006a | 0.01±0.005a | 0.02±0.02a | 3.59±7.16a |
| *Clonostachys* | 3.48±0.76ab | 5.89±3.04a | 4.73±1.37ab | 2.71±0.49b |
| *Aspergillus* | 1.41±0.35a | 1.52±1.06a | 1.16±0.23a | 2.26±1.18a |
| *Talaromyces* | 3.13±0.69a | 3.60±2.28a | 4.33±1.98a | 2.15±0.51a |
| *Trichoderma* | 8.84±14.50a | 2.29±1.69a | 1.89±0.45a | 1.44±0.15a |
| *Leptodiscella* | 0.45±0.51a | 0.09±0.11a | 0.72±0.66a | 1.42±2.01a |
| *Sagenomella* | 1.48±1.13a | 1.51±0.68a | 1.49±0.56a | 1.35±0.70a |
| *Neocosmospora* | 1.18±0.22a | 1.43±0.58a | 1.56±0.34a | 1.30±0.47a |
| *Gibberella* | 0.97±0.38a | 1.00±0.36a | 1.00±0.57a | 1.25±0.22a |
| *Penicillium* | 0.82±0.13ab | 0.83±0.37ab | 0.54±0.23b | 1.09±0.20a |
| *Pseudeurotium* | 1.03±0.26a | 0.89±0.26a | 1.17±0.39a | 1.07±0.33a |
| *Lecythophora* | 0.34±0.21a | 1.19±1.24a | 0.80±0.44a | 1.07±1.21a |
| *Exophiala* | 0.74±0.25a | 0.96±0.43a | 1.07±0.46a | 0.83±0.34a |
| *Chordomyces* | 0.86±0.73a | 1.09±1.55a | 0.89±0.48a | 0.68±0.42a |
| *Gonytrichum* | 0.8±0.46ab | 0.79±0.32ab | 1.48±0.92a | 0.63±0.10b |
| *Pseudaleuria* | 0.63±0.67a | 1.27±0.88a | 1.33±1.82a | 0.53±0.27a |
| *Stilbella* | 0.82±1.07a | 2.64±4.20a | 0.98±1.14a | 0.50±0.39a |
| *Lecanicillium* | 2.04±2.89a | 1.22±1.27a | 1.87±2.94a | 0.49±0.54a |
| *Volutella* | 0.20±0.24a | 0.35±0.49a | 2.48±4.75a | 0.33±0.37a |
| *Psathyrella* | 2.15±4.27a | 0.06±0.10a | 0.02±0.03a | 0.32±0.39a |
| *Chlamydocillium* | 1.37±2.48a | 0.06±0.11a | 0.19±0.35a | 0.12±0.19a |
| *Pyrenochaeta* | 0.09±0.10a | 4.30±8.51a | 0.28±0.42a | 0.11±0.12a |
| *Microscypha* | 1.27±2.53a | 0.04±0.03a | 0.02±0.02a | 0.01±0.01a |

**Table S3** The top 10 species in the betweenness centrality values of the symbiotic network analysis.

| Groups | Bacteria | | | Fungi | | |
| --- | --- | --- | --- | --- | --- | --- |
|  | Phylum | Genus | Betweeness Centrality | Phylum | Genus | Betweeness Centrality |
| CK | Chloroflexi | *g__norank_c__JG30-KF-CM66* | 2365.19575 | Ascomycota | *g__unclassified_p__Ascomycota* | 2555 |
|  | Proteobacteria | *g__norank_c__Alphaproteobacteria* | 1656.337236 | Ascomycota | *Sagenomella* | 2537 |
|  | Actinobacteria | *g__norank_f__Ilumatobacteraceae* | 1397.661488 | Ascomycota | *Setophoma* | 2077.757937 |
|  | Acidobacteria | *Terracidiphilus* | 1178.746549 | Basidiomycota | *Cystofilobasidium* | 2034.845238 |
|  | Proteobacteria | *Sphingomonas* | 1109.456786 | Ascomycota | *Cladosporium* | 1921.480952 |
|  | Proteobacteria | *Anaeromyxobacter* | 1075.626924 | Ascomycota | *Neocosmospora* | 1561.273922 |
|  | Chloroflexi | *g__norank_f__A4b* | 1025.48707 | Ascomycota | *Fusidium* | 1423.535714 |
|  | Chloroflexi | *g__norank_f__AKYG1722* | 961.273322 | Ascomycota | *Taeniolella* | 1372.169048 |
|  | Verrucomicrobia | *ADurb.Bin063-1* | 917.80307 | Ascomycota | *g__unclassified_f__Clavicipitaceae* | 1333.999485 |
|  | Proteobacteria | *Sorangium* | 897.690406 | Ascomycota | *Gibellulopsis* | 1254.714286 |
| CF | Proteobacteria | *mle1-7* | 1555.715599 | Basidiomycota | *g__unclassified_c__Agaricomycetes* | 1973.665286 |
|  | Gemmatimonadetes | *Gemmatimonas* | 1151.77445 | Ascomycota | *Boeremia* | 1963.331952 |
|  | Proteobacteria | *g__unclassified_c__Deltaproteobacteria* | 1147.251667 | Ascomycota | *Gonytrichum* | 1952.571068 |
|  | Proteobacteria | *g__norank_c__Alphaproteobacteria* | 1089.108364 | Ascomycota | *Periconia* | 1927.278552 |
|  | Gemmatimonadetes | *g__norank_f__Gemmatimonadaceae* | 1088.511232 | Ascomycota | *Gibellulopsis* | 1889.657931 |
|  | Armatimonadetes | *g__norank_p__Armatimonadetes* | 1067.156628 | Ascomycota | *Myrmecridium* | 1267.470479 |
|  | Proteobacteria | *g__unclassified_f__Polyangiaceae* | 1037.513032 | Ascomycota | *Paramyrothecium* | 1215.362864 |
|  | Proteobacteria | *g__norank_o__Elsterales* | 1016.740542 | Basidiomycota | *Cutaneotrichosporon* | 1111.369115 |
|  | Actinobacteria | *Streptosporangium* | 985.657638 | Ascomycota | *Acremonium* | 888.14887 |
|  | Bacteroidetes | *g__norank_o__OPB56* | 898.944922 | Ascomycota | *g__unclassified_f__Pezizaceae* | 881.828965 |
| OF | Proteobacteria | *g__norank_f__Diplorickettsiaceae* | 1537.261508 | Mortierellomycota | *g__unclassified_p__Mortierellomycota* | 1095.754047 |
|  | Proteobacteria | *g__Hyphomicrobium* | 1349.17986 | Ascomycota | *g__unclassified_o__Sordariales* | 1087.61826 |
|  | Proteobacteria | *Rhodoplanes* | 971.906822 | Ascomycota | *g__unclassified_f__Arthopyreniaceae* | 1040.641777 |
|  | Proteobacteria | *g__norank_o__CCD24* | 950.223768 | Ascomycota | *Thelonectria* | 1023.450533 |
|  | Proteobacteria | *Cupriavidus* | 944.566625 | Ascomycota | *Setophoma* | 935.698348 |
|  | Actinobacteria | *g__unclassified_o__Gaiellales* | 929.987208 | Ascomycota | *g__unclassified_f__Lasiosphaeriaceae* | 787.522053 |
|  | Chloroflexi | *g__norank_c__Gitt-GS-136* | 908.74532 | Ascomycota | *Dactylonectria* | 715.911632 |
|  | Proteobacteria | *Anaeromyxobacter* | 882.505801 | Ascomycota | *Westerdykella* | 708.480795 |
|  | Actinobacteria | *Microbispora* | 870.910828 | Ascomycota | *Microdochium* | 596.385043 |
|  | Proteobacteria | *alphaI_cluster* | 854.504492 | Ascomycota | *Talaromyces* | 594.799674 |
| OMF | Proteobacteria | *Pseudomonas* | 1737.968769 | Ascomycota | *Metarhizium* | 692.461623 |
|  | Acidobacteria | *g__norank_o__Subgroup_7* | 1607.103537 | Basidiomycota | *Apiotrichum* | 478.280303 |
|  | Chloroflexi | *g__norank_o__SBR1031* | 1333.237844 | Ascomycota | *Acremonium* | 348.158414 |
|  | Bacteroidetes | *g__norank_f__AKYH767* | 1282.030999 | Ascomycota | *Collarina* | 304.949416 |
|  | Proteobacteria | *g__norank_f__BIrii41* | 1185.829983 | Ascomycota | *Fusidium* | 273.564394 |
|  | Actinobacteria | *Mycobacterium* | 1134.52874 | Ascomycota | *Westerdykella* | 180 |
|  | Proteobacteria | *Ellin6067* | 1094.20737 | Ascomycota | *Arachnomyces* | 175.156547 |
|  | Actinobacteria | *Blastococcus* | 1042.10149 | Ascomycota | *g__unclassified_p__Ascomycota* | 175.156547 |
|  | Proteobacteria | *g__norank_f__A21b* | 983.268509 | Ascomycota | *Periconia* | 147.136364 |
|  | Proteobacteria | *g__norank_f__Sandaracinaceae* | 981.727322 | Ascomycota | *Sagenomella* | 141.021248 |

**Figure S1** Schematic diagram of experimental plots divided based on RCBD.


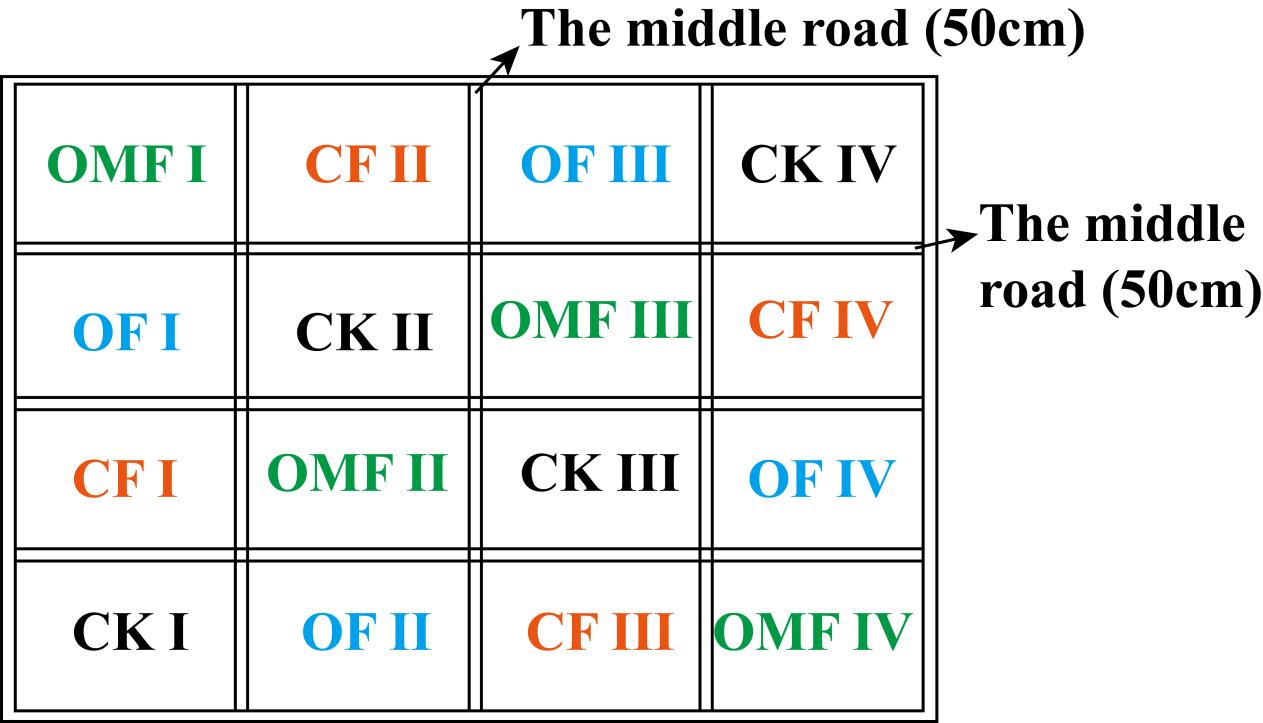


**Figure S2** Modular classification of microbial symbiotic networks.


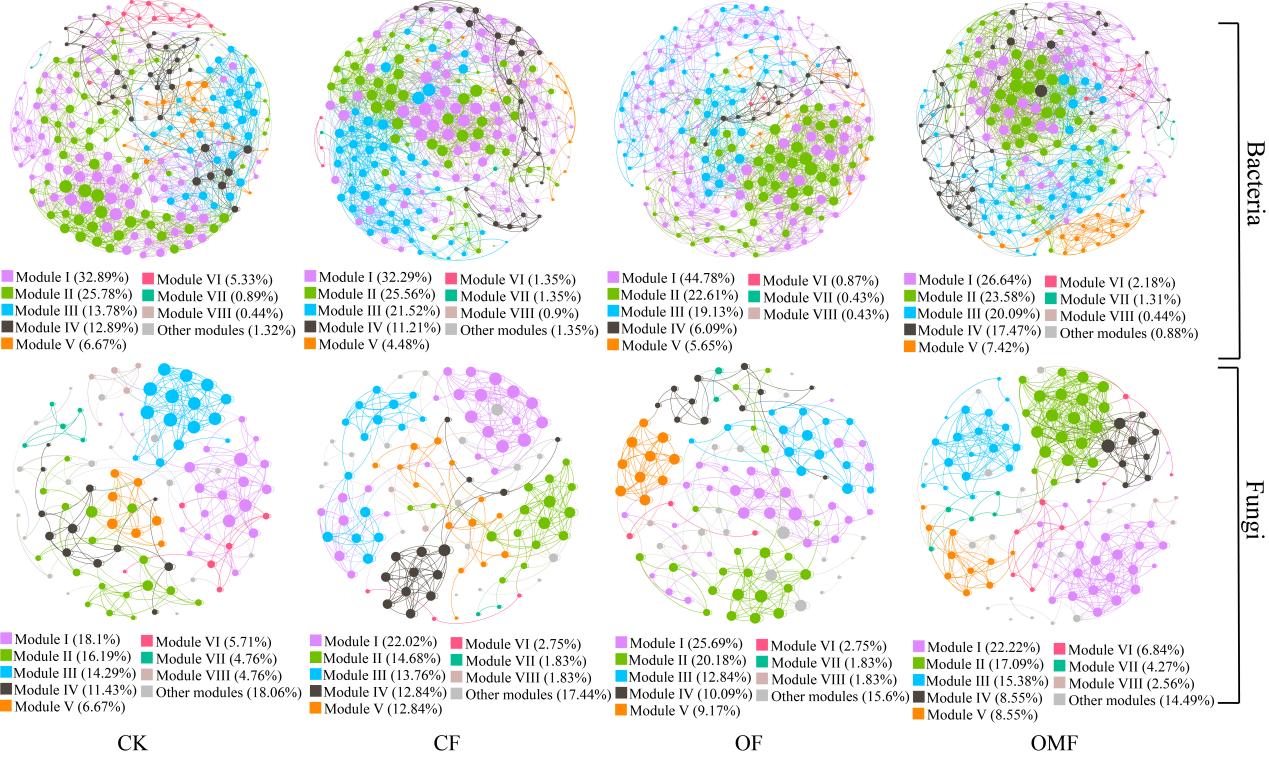


**Figure S3** Relative abundance of AM fungi among symbiotic trophic fungi in different treatment groups. (A) Percentage of AM fungi in total Guild types in each group; (B) Relative abundance of AM fungi in each group at the taxonomic level of family.


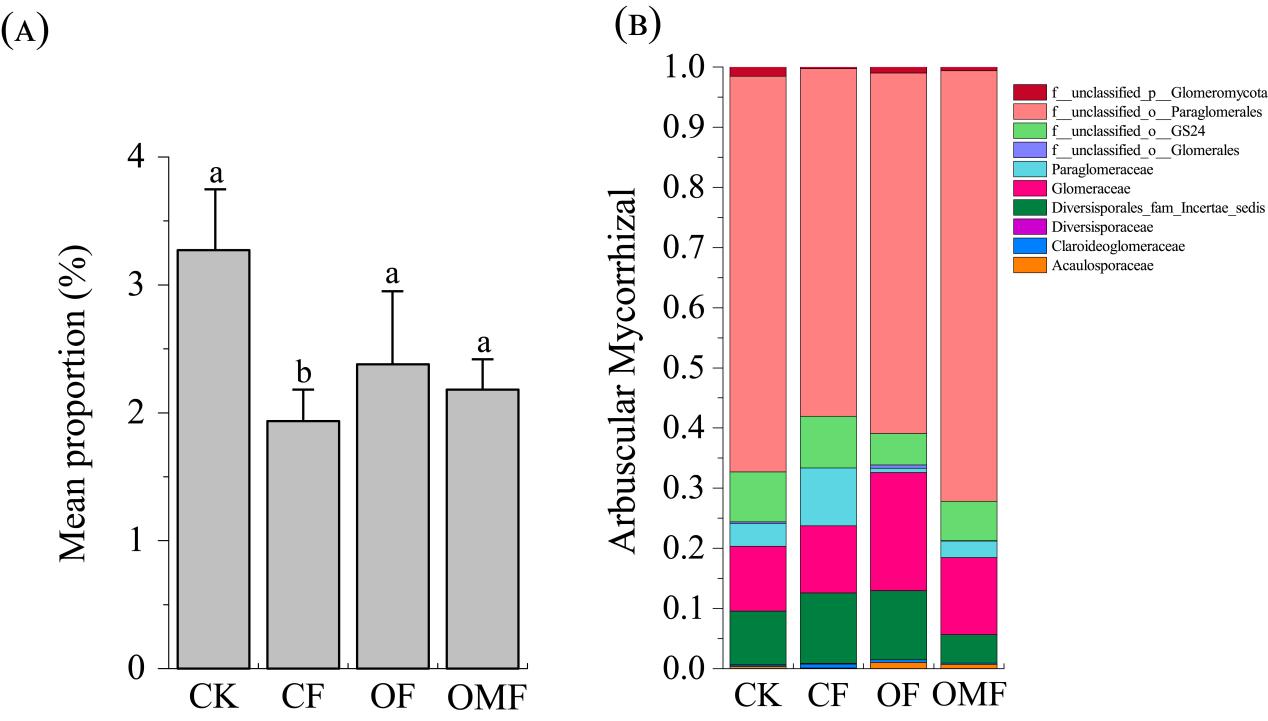

Supplement: Supplementary file 1 [file Data_Sheet_1.docx]
